# A 2015 inventory of embodied carbon emissions for Chinese power transmission infrastructure projects

**DOI:** 10.1038/s41597-020-00662-4

**Published:** 2020-10-01

**Authors:** Wendong Wei, Meng Wang, Pengfei Zhang, Bin Chen, Dabo Guan, Shuai Shao, Jiashuo Li

**Affiliations:** 1 School of International and Public Affairs, Shanghai Jiao Tong University, Shanghai, 200030 China; 2 Business School, University of Shanghai for Science and Technology, Shanghai, 200093 China; 3 Institute of Blue and Green Development, Shandong University, Weihai, 264209 China; 4 College of Engineering, Peking University, Beijing, 100871 China; 5 Department of Earth System Sciences, Tsinghua University, Beijing, 100080 China; 6 School of Business, East China University of Science and Technology, Shanghai, 200237 China

**Keywords:** Climate-change mitigation, Energy supply and demand, Climate-change policy

## Abstract

The spatial mismatch of energy resources and electricity demand in China drives the large-scale construction of power transmission infrastructure, which consumes a large amount of carbon-intensive products. However, a systematic accounting framework for the carbon emissions of power transmission infrastructure has not yet been established. This study for the first time compiles an embodied carbon emissions inventory covering 191 typical power transmission infrastructure projects in China in 2015, including 145 types of alternating current (AC) transmission line projects, 37 typical AC substation projects, 8 typical direct current (DC) transmission line projects and 1 typical DC converter station project. The inventory also shows the detailed inputs of all the projects. These data not only enable a quantitative assessment of the embodied carbon emissions of power transmission infrastructure in China but also provide essential information for climate mitigation policy design in the power sector.

## Abstract (summary)

**Table:** 

Measurement(s)	carbon atom • Emission
Technology Type(s)	digital curation
Factor Type(s)	terrain type • voltage class
Sample Characteristic - Environment	electric power system
Sample Characteristic - Location	China

Machine-accessible metadata file describing the reported data: 10.6084/m9.figshare.12911645

## Background & Summary

The spatial mismatch between China’s energy centres and load centres has led to China’s large-scale power transmission infrastructure construction^1,2^. In 2001, the Chinese government launched the West-East Power Transmission project^3^, which triggered the large-scale construction of transmission infrastructure. From 1990 to 2015, the total length of 220 kV and above transmission lines increased from 8.43 × 10^4^ km to 6.83 × 10^5^ km, making China the country with the largest transmission infrastructure in the world. Notably, in recent years, Chinese government attaches great importance to the construction of ultra-high voltage (UHV) power grids, which will lead to the acceleration of domestic power transmission infrastructure construction. In addition, the Global Energy Interconnection strategy proposed by the Global Energy Interconnection Development and Cooperation Organization (GEIDCO)^4^ has also received support from Chinese government and many other countries in the world, and will promote the construction of power transmission infrastructure worldwide^5,6^.

The construction of power transmission infrastructure can directly lead to carbon emissions from the consumption of gasoline, diesel, etc.^7^, and can also indirectly lead to carbon emissions through the inputs of building materials (cement, steel, etc.) and electric equipment (cables, transformers, hanging wire fittings, etc.)^8–11^. Researchers have calculated greenhouse gas (GHG) emissions of transmission infrastructure in European regions^12^, countries^13,14^ and Northern Europe^15^ and verified that transmission infrastructure construction has a great impact on climate. However, the impact of the world’s largest power transmission infrastructure remains unknown. Moreover, there were limitations in the previous studies. First, in these studies, only data of raw materials were collected to calculate GHG emissions caused by construction processes^16^, and data of tools, vehicles, office equipment and other materials used in construction processes were not statistically complete. Second, these studies were based on the production and construction data of developed regions such as Europe and provide limited guidance for large-scale transmission infrastructure construction in developing countries.

To have a clear understanding of the carbon emissions caused by China’s transmission infrastructure construction, this study builds an embodied carbon emissions inventory of power transmission infrastructure in China in 2015 by combining process analysis and input-output (IO) analysis. The dataset described here includes more than 10,000 inputs and their emissions for 191 typical power transmission infrastructure projects in China, involving projects in six different terrains and six different voltage classes. All the data have been uploaded to the open-access online dataset figshare (figshare.com) for free download.

The dataset can be used, including but not limited to, in the following ways:

1. Analysing the changes in the embodied carbon emissions of China’s transmission infrastructure over a period
2. Evaluating the structure of carbon emissions caused by transmission infrastructure in different regions of China and optimizing the design of transmission infrastructure
3. Incorporating carbon emissions into the construction feasibility assessment of the project to achieve a more environmentally friendly construction process

## Methods

### Input data collection

The input inventory for transmission line, substation and converter station projects is derived from the General Cost of Power Transmission and Distribution Project of the State Grid Corporation of China (GCPT)^17–27^, which has more than 10 sub-volumes. The sub-volumes of 220 kV, 330 kV, 500 kV, and 750 kV AC substations and 220 kV, 330 kV, 500 kV, and 750 kV AC transmission lines are used. In addition, for UHV parts, the sub-volumes of ± 800 kV DC converter stations and transmission lines and 1000 kV AC substations and transmission lines are also collected. In each volume, there are different construction cases and input cost data for six different terrains (including flatland, hill, mountain, high mountain, desert, and river swamp). This series of books reports the general cost of China’s power transmission infrastructure and can be used by all power system designers, as well as professionals engaged in power engineering planning, management, construction and installation, and can also be used as a reference for researchers and students of relevant majors in universities.

All the input data were extracted by one member of our group and then confirmed by another member to ensure the accuracy of the extracted data^28^. We aggregate the same type inputs with different voltage class into a same classification. For example, 1000 kV power devices, 500 kV power devices, 110 kV power devices and other power devices were all integrated into the input of transmission and distribution equipment. To ensure the accuracy of data merging, a third member of the group checked the data again.

### Embodied carbon emission intensity calculation

First, we built a direct emission inventory from EXIOBASE^29,30^, and then adopted an environmentally extended input-output analysis (EEIOA)^31–33^ to calculate the embodied emissions intensities^34^, which are expressed as:

1 $$e=E{(\widehat{X}-A)}^{-1}$$

where *e* is a 1 × N matrix that represents sectoral embodied carbon emissions intensities; *E* is a 1 × N matrix of sectoral direct carbon emissions in year *t*; $\widehat{X}$ represents the diagonal matrix of sectoral total output; and *A* represents the intermediate input matrix.

Input-output (IO) tables from EXIOBASE were used to calculate the embodied carbon emissions intensities. IO tables from EXIOBASE enable us to differentiate China’s domestic products and imports. It is worth noting that when using single regional IO tables to calculate embodied carbon intensities, there is an assumption that the embodied carbon emissions intensities of domestic products and imports are the same, and this assumption may lead to great uncertainty^35^, as China’s energy structure and industrial technology vary significantly from those in other countries. By contrast, IO tables from EXIOBASE clearly differentiate domestic products and imports, thus enabling us to obtain a more accurate carbon intensity^36^.

### Embodied carbon emissions calculation

The EXIOBASE IO table includes 163 sectors and has a higher sector resolution than the China IO table, which contains 42 sectors. The higher the sector resolution that an IO table has, the more accurate the intensity data could be, as aggregation of sectors will lead to biased results^36^. We followed these sector definitions to construct our emission inventories. It is worth noting that the monetary unit of the original input inventories is CNY, while the monetary unit of EXIOBASE is EUR. Therefore, the average exchange rate in 2015 was used for currency conversion^37^. Like the input data collection, one of our team members matched the inputs with IO table sectors, and another member verified the results. The emissions can be calculated as:

2 $$C{E}_{a}={V}_{a}\times {E}_{a}$$

where *CE*_*a*_ represents the embodied carbon emissions of Sector a, *V*_*a*_ represents the input quantity of Sector *a* and *e*_*a*_ represents the embodied emission intensity of Sector *a*.

## Data Records

The database is publicly available from Figshare^38^.

(1) Part 1. 1 table of the corresponding relationship among the original data, initial classification of inputs and IO sectors.

- **Original data classification** (column A): The classification of the original data in the book.
- **Detailed input sectors** (column B): When our team extracted the data, we aggregated some data according to the sector resolution used in this study.
- **IO sector** (column C): The sectors in the EXIOBASE IO table.

(2) Part 2. 191 tables of the inventories of the inputs and embodied carbon emissions of 191 typical projects (145 types of AC transmission line projects, 37 typical AC substation projects, 8 typical DC transmission line projects and 1 typical DC converter station project).

- **Detailed input sectors** (column A): Same as the detailed input sectors introduced above.
- **Monetary value of CNY** (column B and column C): Value of each input in CNY.
- **Exchange rate between EUR and CNY** (column D): Average exchange rate of the year for converting CNY to EUR.
- **Monetary value of EUR** (column E and column F): Value of each input in EUR.
- **Sectors and embodied emission intensities** (column G and column H): The sectors in the EXIOBASE IO table and the embodied carbon emission intensities calculated by EEIOA.
- **Embodied emissions** (column I): The total embodied emissions and emissions of each input.

(3) Part 3. 1 table summarizing the embodied carbon emissions from transmission line projects.

- **Project code** (column A): The codes of different typical projects.
- **Terrain type** (column B): The terrain of the project. The transmission line projects involve six different terrains (including flatland, hill, mountain, high mountain, desert, and river swamp).
- **Voltage classes** (column C): The voltage class of the project. Our research object is transmission infrastructure above 220 kV in China, including 220 kV, 330 kV, 500 kV, 750 kV, ± 800 kV and 1000 kV.
- **Circuit style** (column D): The circuit style of the transmission lines. There are two types of transmission lines for all voltage classes: single circuit and double circuit.
- **Transmission length** (column F): The distance of a typical project. The length of most typical projects is 50 kilometres. However, due to terrain limitations, the lengths of some typical projects are slightly different.
- **Embodied emissions**: The total embodied carbon emissions of the project.

(4) Part 4. 1 table summarizing the embodied carbon emissions from substation and converter station projects. The details are the same as those in the tables above.

We can see the differences in total embodied emissions between projects by analysing those emissions from transmission line, substation and converter station projects. Among the transmission line projects, projects 10GB4S, 10GB3H and 10GB4Q are the three typical projects with the highest embodied carbon emissions, which are 128 kt, 116 kt and 110 kt, respectively. It can be clearly seen that projects with a high voltage class and double circuit caused more emissions. The same features can also be found in substation projects. Projects 10A-2, 10B-1 and 7A-1 emit the most embodied carbon emissions of all substation projects, with values of 325 kt, 305 kt and 115 kt, respectively. Compared with the substations, the 800 kV converter station produces more embodied carbon emissions, 2.7 times that of project 10A-2. The main structure of a converter station includes a valve hall, converter transformer, AC switching plant, smoothing reactor, filter and reactive power compensation equipment. These structures need large amounts of metal and building materials, which are carbon-intensive products.

**Fig. 1:**
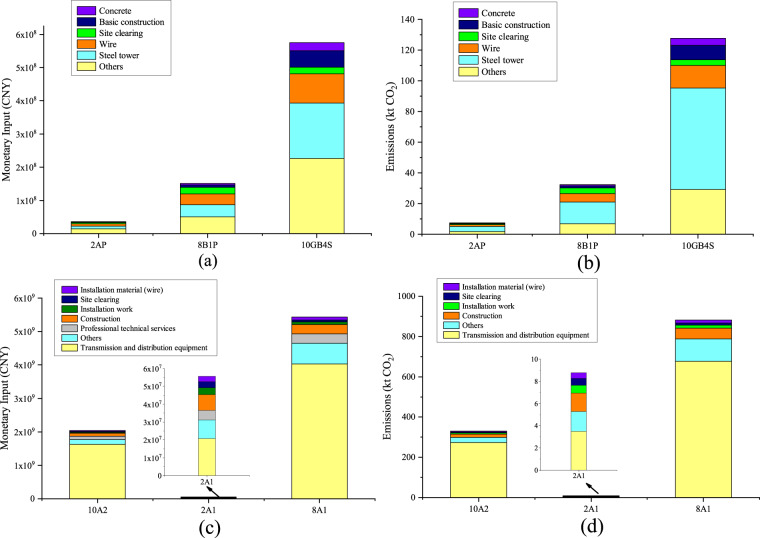
The monetary inputs and emissions of partial transmission infrastructure projects. (**a**: The monetary inputs of transmission line projects 2AP, 8B1P and 10GB4S; **b**: The emissions of transmission line projects 2AP, 8B1P, and 10GB4S; **c**: The monetary inputs of substation projects 2A1 and 10A2 and converter station project 8A1; **d**: The emissions of substation projects 2A1 and 10A2 and converter station project 8A1).

The proportions of the inputs within each typical project show different characteristics. Here, we choose substation project 2A1 with the lowest emissions, substation project 10A2 with the highest emissions, transmission line project 2AP with the lowest emissions, transmission line project 10GB4S with the highest emissions, and the 800 kV converter station for analysis (Fig. 1). In typical project 2A1, the monetary proportion and emission proportion of equipment input (transmission and distribution equipment) are the highest, accounting for 37.32% of the project budget and 39.74% of the total emissions, respectively. The second-largest proportions are due to construction engineering, which accounted for 15.85% of the budget and caused 18.82% of emissions, respectively. However, unlike the equipment and construction industries, the embodied emissions intensity of services is low, and professional technical services represent only 2.01% of carbon emissions and 9.68% of the budget. With increasing voltage class, the budget proportions of construction, installation and other parts gradually decrease, while the budget proportion of equipment increases. In typical project 10 A2, the budget proportion and emission proportion of equipment input (transmission and distribution equipment) are 79.67% and 82.80%, respectively. Professional technical services, ranked second in budget share (5.18%), caused 1.05% of emissions. The budget and emission structures of the converter station are similar to those of typical project 10A-2. Equipment input (transmission and distribution equipment) accounts for the vast majority of the budget and emissions, 74.18% and 76.90%, respectively. Construction creates 6.03% of emissions and uses 5.21% of the budget, constituting the second-largest emission sector. The input budget and emission structures of 2AP and 10GB4S are very similar. Steel towers, wires and construction site clearing represent the top three aspects of the budget and emissions, with the former two accounting for approximately 20% and the latter accounting for approximately 10%.

## Technical Validation

### Uncertainties

The input inventories and carbon emission intensities are two main reasons leading to uncertainties in this study. According to the equations in the uncertainty analysis, the key parameter for the simulation is the relative standard deviation (RSD), also known as the coefficient of variation (CV). Here, we assume a CV of 10% for the various input inventories. For the carbon emission intensities, our study is based on EXIOBASE 3.3. However, no official uncertainty information is available for EXIOBASE. Therefore, we use other CVs of the proxy data based on Hertwich and Peters^39^. Detailed CVs and their specifications are listed in Table S1. It should be noted that the carbon emission inventory for China has the largest impact on overall uncertainties in this study, and a specific estimation of the CVs for China’s carbon emission inventory is conducted based on the China Emission Accounts and Datasets^40^ (CEADs). The CEADs provide CVs for energy consumption and corresponding emission factors for various sectors^41–44^ (shown in Tables S2 and S3). Consequently, we use such information to generate the CVs of the carbon emission inventory for various sectors.

Then, a Monte Carlo perturbation was adopted to conduct at least 10,000 simulations for the given source data, based on which the probability distributions of errors in individual source input parameters could be obtained.

The results show that the maximum uncertainty range appears in typical project 10A-2 and is (−18.05%, +21.06%). The 10 projects with the largest uncertainty ranges among the transmission line and substation projects are shown in Table 1. Other uncertainty results are shown in Table S4.

**Table 1:** The uncertainties of parts of typical projects.

	Project code	95% CI (−)	95% CI (+)
Transmission line projects	2FG	14.85%	16.48%
	2GS	14.73%	16.43%
	2FS	14.70%	16.45%
	5HQ	14.63%	16.47%
	5EP	14.36%	16.74%
Substation projects	10A2	18.05%	21.06%
	7A1	17.55%	20.29%
	10B1	16.86%	19.87%
	5A-3(750 MVA)	16.90%	19.76%
	5A4	16.24%	19.96%
Converter station projects	8A-1	17.58%	21.07%

### Comparison with the existing studies

In order to validate our dataset, we compared our study with existing studies using life cycle assessment (LCA)^8,10,14,16,45^. The input inventory in the current study covers products such as temporary facilities, fixed machinery cost, construction tools and appliances, which are integral parts for transmission infrastructure construction. However, these inputs were not considered by the LCA based studies, thus inevitably leading to underestimation for embodied carbon emissions of transmission infrastructure. Moreover, the present study used IO to calculate the embodied carbon emission intensities, which can avoid the endless trace and truncation error problems inherent in LCA^46,47^. Therefore, our study provides a more comprehensive and accurate carbon emission accounting for China’s transmission infrastructure.

## Supplementary information

Supplementary tables

## Data Availability

Matlab program is used to generate the hybrid method calculation and Mote Carlo simulation. The full custom MATLAB script is provided on the open-access online dataset figshare^48^ and GitHub (https://github.com/conanbean/A-2015-inventory-of-embodied-carbon-emissions-for-Chinese-power-transmission-infrastructure-projects.git). The carbon emissions are calculated by multiplying the various inputs of the project by the corresponding embodied carbon intensity. The sectoral embodied carbon intensities in China are calculated by using EEIOA (see algorithm in Method), based on EXIOBASE database. And then, the stochastic modelling is adopted to carry out Monte Carlo simulation in terms of the standard deviation (SD). We define the order of magnitude of each source data *x* as lg *x*, then the perturbation of *x* (denoted as *x*^*Φ*^) is $lg{x}^{\Phi }\approx lg(x+dx)=lgx+lg((x+dx)/x)=lgx+lg(1+Rx)$. The *Rx* represents the relative SD, which is also named as coefficient of variation (CV). Consequently, the perturbation be conducted 10000 times for every raw data, including input inventory, carbon emission inventory, and each item in MRIO table, to obtain the SD of the embodied carbon emissions for each project.
