# Supplementary material for: A 2015 inventory of embodied carbon emissions for Chinese power transmission infrastructure projects
Source: Sci Data. 2020 Oct 1;7:318. doi: 10.1038/s41597-020-00662-4 (PMC7530655; doi:10.1038/s41597-020-00662-4)
Supplement: Supplementary file 1 — Supplementary tables [file 41597_2020_662_MOESM1_ESM.pdf]

Table S1. Detailed CVs and their specifications.

Parameter	Description and specification	CVs
Emissions inventory	CO_2 OECD	10%
	CO_2 non-OECD	20%
	CO_2 China	Own estimation
MRIO table	input-output coefficients	1-50%
	consumption matrix	10%

Table S2. The CVs of sectoral energy consumption for China

Sectors	CVs
Electricity generation sector[26]	5%
Other industry and construction sector[27]	10%
Transportation sector[28]	16%
Primary industry[29]	30%

Table S3. Emission factors by different sources and corresponding CVs for China

Energy sources	Emission factors (mean)	CVs
Raw Coal	2.05	18%
Clean Coal	2.35	11%
Other Washed Coal	1.96	26%
Briquettes	1.98	18%
Coke	2.86	3%
Coke Oven Gas	9.24	15%
Other Gas	6.14	33%
Other Coking Products	3.12	23%
Crude Oil	3.07	1%
Gasoline	3.05	2%
Kerosene	3.11	1%
Diesel Oil	3.15	1%
Fuel Oil	3.11	2%
LPG	3.06	8%
Refinery Gas	2.87	14%
Other Petroleum Products	3.08	4%
Natural Gas	2.08	5%

Table S4a. The uncertainties of transmission line projects

Project code	95% CI (-)	95% CI (+)
2AG	14.13%	15.93%
2AH	14.34%	16.53%
2AP	14.53%	16.55%
2AQ	14.26%	16.32%
2AS	14.04%	16.54%
2BG	14.14%	16.52%
2BH	14.40%	16.62%
2BP	14.37%	16.41%
2BQ	14.06%	16.03%
2BS	14.23%	16.79%
2CG	14.46%	16.45%
2CH	14.15%	15.95%
2CP	14.29%	16.04%
2CQ	13.85%	16.61%
2CS	14.13%	15.93%
2DG	14.19%	15.64%
2DH	13.86%	15.93%
2DP	14.24%	16.06%
2DQ	14.20%	16.54%
2DS	14.20%	15.93%
2EH	13.97%	16.33%
2EP	14.42%	16.26%
2EQ	14.41%	16.54%
2FG	14.85%	16.48%
2FH	14.47%	16.58%
2FP	14.20%	15.79%
2FQ	14.04%	16.22%
2FS	14.70%	16.45%
2GG	14.08%	15.97%
2GH	14.34%	15.85%
2GP	14.09%	16.20%
2GQ	14.12%	16.32%
2GS	14.73%	16.43%
2HG	14.25%	16.41%
2HH	14.31%	16.31%
2HP	14.34%	16.56%
2HQ	14.34%	16.50%
2HS	14.06%	16.38%

Table S4a. The uncertainties of transmission line projects

Project code	95% CI (-)	95% CI (+)
2IH	14.43%	16.32%
2IP	14.16%	16.18%
2IQ	13.92%	16.58%
3AP	14.09%	16.48%
3BG	13.90%	16.50%
3BM	14.38%	16.21%
3BP	14.27%	16.14%
3BS	14.10%	16.04%
3CG	14.04%	16.26%
3CM	14.35%	16.35%
3CQ	14.44%	16.26%
3CS	14.21%	16.17%
3DG	14.24%	16.38%
3DM	13.93%	16.04%
3DQ	14.25%	16.10%
3DS	14.39%	16.40%
5AG	14.23%	15.87%
5AH	14.52%	16.25%
5AP	13.82%	16.00%
5AQ	14.50%	16.34%
5AS	14.26%	16.08%
5BG	14.38%	15.84%
5BH	13.86%	15.60%
5BP	14.21%	16.26%
5BQ	13.86%	16.32%
5BS	13.98%	15.88%
5CG	14.36%	16.22%
5CH	14.09%	16.20%
5CP	14.18%	16.16%
5CQ	14.22%	16.42%
5CS	14.28%	16.80%
5DG	14.43%	16.18%
5DH	13.98%	15.86%
5DP	14.25%	16.27%
5DQ	14.01%	16.14%
5DS	13.80%	15.30%
5EG	14.08%	16.37%
5EH	14.46%	16.59%

Table S4a. The uncertainties of transmission line projects

Project code	95% CI (-)	95% CI (+)
5EP	14.36%	16.74%
5EQ	14.23%	15.86%
5ES	14.03%	16.35%
5FG	14.11%	16.28%
5FH	13.81%	16.08%
5FP	14.26%	16.35%
5FS	14.00%	15.64%
5GG	14.01%	16.17%
5GH	13.98%	16.75%
5GP	14.29%	16.20%
5GQ	14.32%	16.10%
5GS	14.14%	15.91%
5HG	13.96%	16.38%
5HH	13.97%	16.20%
5HP	14.13%	15.82%
5HQ	14.63%	16.47%
5HS	14.35%	16.14%
5IH	14.37%	16.52%
5IP	14.27%	16.28%
5IQ	14.08%	15.79%
7A1G	14.36%	16.00%
7A1H	14.28%	16.17%
7A1M	14.00%	16.09%
7A1P	14.16%	16.35%
7A1Q	14.11%	16.29%
7A1S	14.27%	16.08%
7A3G	14.18%	16.22%
7A3M	13.65%	16.01%
7A3P	13.94%	16.21%
7A3Q	14.29%	16.26%
7A3S	13.93%	15.89%
7A5G	14.43%	16.04%
7A5M	14.25%	16.41%
7A5P	14.07%	16.39%
7A5Q	13.73%	15.71%
7A5S	14.02%	15.92%
7B1G	13.83%	16.01%
7B1P	14.22%	15.87%

Table S4a. The uncertainties of transmission line projects

Project code	95% CI (-)	95% CI (+)
7B1Q	13.91%	16.28%
7B1S	14.28%	16.38%
7B2G	14.09%	16.19%
7B2P	13.70%	15.88%
7B2Q	14.03%	16.20%
7B2S	14.26%	15.90%
7C2G	13.92%	16.64%
7C2H	14.11%	16.00%
7C2M	14.08%	16.30%
7C2P	14.22%	16.29%
7C2Q	14.26%	15.73%
7C2S	13.80%	16.20%
7D1G	13.81%	15.80%
7D1M	14.06%	16.22%
7D1P	14.11%	16.34%
7D1Q	14.07%	16.35%
7D1S	14.17%	16.41%
10A2G	14.38%	15.96%
10A2S	13.96%	16.16%
10GB1H	14.04%	16.08%
10GB1P	14.19%	16.10%
10GB1Q	14.04%	15.90%
10GB2H	14.27%	16.36%
10GB2P	14.21%	16.58%
10GB2Q	14.09%	15.81%
10GB2S	14.41%	16.17%
10GB3H	14.16%	16.61%
10GB3P	14.42%	16.26%
10GB3Q	14.33%	16.41%
10GB4Q	14.09%	15.90%
10GB4S	14.34%	16.26%
8B1G	14.02%	16.28%
8B1H	14.15%	16.93%
8B1P	14.21%	16.24%
8B1Q	14.12%	16.05%
8B1S	13.82%	15.84%
8B3Q	14.23%	15.85%
8B3S	14.12%	16.16%
8B6G	14.14%	16.21%

Table S4b. The uncertainties of substation projects

Project code	95% CI (-)	95% CI (+)
2A1	16.05%	19.49%
2A3	15.60%	18.68%
2A5	16.18%	18.97%
2A7	16.03%	18.83%
2A8	16.36%	18.60%
2B1	15.92%	18.93%
2B2	15.96%	17.55%
2B3	15.86%	18.41%
2B4	15.94%	18.50%
2B5	16.09%	18.76%
3A1	16.28%	19.26%
3C1	16.18%	18.49%
3C2	15.95%	17.72%
3D1	16.13%	18.66%
3D2	16.09%	18.57%
5A-1(750MVA)	16.28%	19.24%
5A-1(1000MVA)	16.65%	19.40%
5A2	16.65%	19.20%
5A-3(750MVA)	16.90%	19.76%
5A-3(1000MVA)	16.65%	19.22%
5A4	16.24%	19.96%
5B1	16.47%	18.97%
5B2	16.39%	19.03%
5B3	16.36%	18.95%
5C1	15.96%	18.47%
5C2	16.12%	18.26%
5C3	16.11%	18.57%
5D1	15.93%	18.51%
5D2	16.08%	18.32%
5D3	16.08%	18.81%
7A1	17.55%	20.29%
7C1	16.56%	19.06%
7C2	16.25%	19.01%
7C3	16.36%	19.38%
7C4	16.55%	18.59%
10A2	18.05%	21.06%
10B1	16.86%	19.87%

Table S4c. The uncertainties of converter station projects

Project code	95% CI (-)	95% CI (+)
8A-1	17.58%	21.07%
